# Steering health through uncertainty: leadership and transformation in the WHO European Region

**DOI:** 10.1093/eurpub/ckaf267

**Published:** 2026-01-23

**Authors:** Hans Kluge, Martin McKee

**Affiliations:** WHO Regional Office for Europe, UN City Marmorvej, Copenhagen, Denmark; European Observatory on Health Systems and Policies, London School of Hygiene Tropical Medicine, London, United Kingdom

The WHO European Region continues to face an accumulation of intersecting crises that test the resilience of societies and health systems. At the same time, longstanding challenges, from noncommunicable diseases to rising mental health needs, persist with undiminished force. As WHO/Europe launches its Second European Programme of Work 2026–2030 (EPW 2.0) [1], there is an opportunity to draw lessons from recent years while renewing a strategic commitment to leadership, transformation, and partnership. This commentary reflects on the challenges ahead and highlights how strengthened public health leadership, supported by innovation, equity, and trust, will be instrumental in advancing health for all in an era of uncertainty.

The WHO Regional Office for Europe serves a remarkably diverse Region of 53 Member States, spanning multiple political, economic, and demographic contexts. The breadth of this diversity shapes both the challenges countries face and the leadership required to address them. While acute crises, such as conflict, environmental disasters, and large-scale population movements, demand immediate action, these cannot overshadow the persistent, deeply rooted threats that weigh heavily on population health.

Tobacco use remains one of the most visible examples. Smoking rates continue to vary dramatically across countries [2], and the rapid rise of vaping among young people introduces new challenges [3]. Meaningful progress will require confronting the wider commercial determinants of health and resisting pressures that undermine public policies proven to reduce harm.

Noncommunicable diseases remain the leading cause of death and disability, yet they also highlight how the region provides natural laboratories for policy learning. Countries that have invested in public health, prevention, and strong primary health care achieve clear benefits, reminding us that health improvements are possible even in difficult contexts when leadership is strategic and consistent.

Mental health has also emerged as an urgent priority. Millions in the Region live with mental health conditions, often receiving insufficient care. Initiatives such as the Pan-European Mental Health Coalition have helped elevate awareness, but much remains to be done [4]. This is particularly true for children and adolescents, where there are major gaps in service quality, shortages of specialised workers, and a lack of system-wide leadership structures. These concerns resonate with contributions to this Special Issue, which highlight the critical role of leadership in strengthening child and youth mental health services and building a future-ready workforce.

Demographic change, particularly ageing, adds a further layer of complexity. With smaller working-age populations and increasing care needs, many countries face simultaneous pressures on health financing, workforce availability and long-term care provision. Yet this, too, creates opportunities for mutual learning, especially in healthy ageing, community-based care, and new models of service delivery [5].

## The crises that shape the present and the future

The effects of the COVID-19 pandemic continue to reverberate through Long COVID [6], educational disruption, and growing inequities. The report of the Pan European Commission on Health and Sustainable Development, chaired by Mario Monti [7], underscored the need for preparedness, solidarity and decisive leadership, demonstrating how delays in decision-making can have vast consequences.

Other crises have added to this burden. Recent outbreaks, including Mpox, vaccine-derived poliovirus, and antimicrobial resistance, remind us that infectious threats remain ever-present. Migration pressures driven by conflict, instability and climate stress [8] continue to challenge systems already struggling with workforce shortages [9]. The mass displacement triggered by the war in Ukraine has placed unprecedented pressure on neighbouring countries [10]. These events illustrate how health systems must navigate humanitarian, epidemiological and economic uncertainties simultaneously.

Climate change, increasingly experienced through heatwaves, floods and wildfires, has now become one of the most serious long-term threats to public health in Europe [11]. As temperatures continue to rise, impacts on health, infrastructure and food systems will intensify. The need for mitigation, adaptation and resilient health systems is now indisputable.

## EPW 2.0: a shared agenda for transformation

Against this backdrop, EPW 2.0 provides a renewed strategic direction for the Region from 2026 to 2030. Its vision reinforces commitments to equity, resilience, preparedness, and innovation, while sharpening the focus on the areas where swift and collective action is most needed.

EPW 2.0 elevates several Special Initiatives, including mental health, health and care workforce strengthening, digital health, climate and health, immunization, and quality of care. Each responds to an urgent regional gap; together, they form an integrated agenda for strengthening public health leadership at every level.

Notably, EPW 2.0 emphasises trust, both in institutions and between them [12]. Without trust, even the most evidence-based policies struggle to succeed. The pandemic showed that transparent communication, people-centred policies, and meaningful community engagement are indispensable to the performance of the health system. Papers in this Special Issue address this point, offering insights into indicators, workforce development, and youth-led approaches that collectively build accountability and foster trust.

## Leadership at the centre of transformation

Across the challenges described above, one theme emerges consistently: leadership matters.

Effective leadership is not limited to senior officials. It must be cultivated across the health ecosystem, from national authorities to local managers, frontline clinicians, youth leaders, researchers and communities. This broader understanding is reflected in the contributions to this Special Issue, which range from leadership in quality improvement and patient safety, to the development of public health indicators, to the leadership challenges facing young researchers across the Region.

Leadership is needed to champion quality of care, particularly at a time when services face increasing demand, workforce strain and complex system-wide constraints. Leadership is necessary to build teams that can operate safely under pressure and to design models of care that reflect what patients, especially children, adolescents and their families, value most. It also plays a crucial role in nurturing the next generation of public health professionals, ensuring they have the competencies, confidence and opportunities required to steer systems through future uncertainties.

The Region has already demonstrated that when strong leadership aligns with purpose and partnership, rapid progress is possible. Whether confronting the commercial determinants of health, coordinating regional surveillance, or integrating digital tools into service delivery, leadership provides the direction needed for countries to move forward together.

## Looking ahead

Europe is navigating a period of profound uncertainty. Yet this moment also offers an extraordinary opportunity for renewal. EPW 2.0 sets out a path for building healthier, more resilient and more equitable societies, but its success will depend on the leadership capacity across countries and institutions.

Ultimately, no single organisation or sector can meet today’s challenges alone. Collective leadership, rooted in evidence, grounded in solidarity, and focused on people, is essential. The Special Issue to which this editorial contributes highlights the range of perspectives, experiences and innovations already emerging across the Region. These insights reinforce a simple but powerful message: by investing in public health leadership at all levels, Europe can steer through uncertainty and shape a healthier future for all. Part A of this supplement is available at https://academic.oup.com/eurpub/issue/35/Supplement_2.

Conflict of interest: HK is the Regional Director of the WHO European Region. MM is co-director of the European Observatory, which is co-funded by WHO EURO.

## Data Availability

No new data were generated or analysed in support of this research.
